# Performance of PES/LSMM-OGCN Photocatalytic Membrane for Phenol Removal: Effect of OGCN Loading

**DOI:** 10.3390/membranes8030042

**Published:** 2018-07-11

**Authors:** Noor Elyzawerni Salim, Nor Azureen Mohamad Nor, Juhana Jaafar, Ahmad Fauzi Ismail, Takeshi Matsuura, Mohammed Rasool Qtaishat, Mohd Hafiz Dzarfan Othman, Mukhlis Abdul Rahman, Farhana Aziz, Norhaniza Yusof

**Affiliations:** 1 Advanced Membrane Technology Research Centre (AMTEC), Universiti Teknologi Malaysia, Skudai 81310, Johor, Malaysia; elyza.werni@gmail.com (N.E.S.); zureen.mnor@gmail.com (N.A.M.N.); afauzi@utm.my (A.F.I.); hafiz@petroleum.utm.my (M.H.D.O.); mukhlis@petroleum.utm.my (M.A.R.); farhanaaziz@utm.my (F.A.); norhaniza@utm.my (N.Y.); 2 Faculty of Chemical and Energy Engineering (FCEE), Universiti Teknologi Malaysia, Skudai 81310, Johor, Malaysia; 3 Industrial Membrane Research Laboratory, Department of Chemical Engineering, University of Ottawa, Ottawa, ON K1N 6N5, Canada; matsuura@eng.uottawa.ca; 4 Chemical Engineering Department, The University of Jordan, Amman 11942, Jordan; mohammed.qtaishat@gmail.com

**Keywords:** hydrophilic surface modifying macromolecules, oxygen-doped graphitic carbon nitride, photocatalytic, hybrid membrane, phenol

## Abstract

In designing a photocatalytic oxidation system, the immobilized photocatalyst technique becomes highly profitable due to its promising capability in treating organic pollutants such as phenols in wastewater. In this study, hydrophilic surface modifying macromolecules (LSMM) modified polyethersulfone (PES) hybrid photocatalytic membranes incorporated with oxygenated graphitic carbon nitride (OGCN) was successfully developed using phase inversion technique. The effectiveness of the hybrid photocatalytic membrane was determined under different loading of OGCN photocatalyst (0, 0.5, 1.0, 1.5, 2.0, and 2.5 wt%). The best amount of OGCN in the casting solution was 1.0 wt% as the agglomeration did not occur considering the stability of the membrane performance and morphology. The highest flux of 264 L/m^2^·h was achieved by PES/LSMM-OGCN1.5wt% membrane. However, the highest flux performance was not an advantage in this situation as the flux reduced the rejection value due to open pores. The membrane with the highest photocatalytic performance was obtained at 1.0 wt% of OGCN loading with 35.78% phenol degradation after 6 h. Regardless of the lower rejection value, the performance shown by the PES/LSMM-OGCN1.0wt% membrane was still competent because of the small difference of less than 1% to that of the PES/LSMM-OGCN0wt% membrane. Based on the findings, it can be concluded that the optimisation of the OGCN loading in the PES hybrid photocatalytic membrane indeed plays an important role towards enhancing the catalyst distribution, phenol degradation, and acceptable rejection above all considerations.

## 1. Introduction

There are several established processes that have been applied for wastewater treatment biologically, physically, or chemically [1]. Each of the processes offer different separation mechanisms with certain advantages and limitations. Following the water treatment issue, the membrane separation technology has become one of the trusted methods in removing contaminants for many years and its capability is slowly taking over the tasks of using conventional chemical and biological wastewater treatments. Membrane technology is a flexible separation method and can be designed as per the needs of industry. Polymeric membrane is considered a preferable separation medium to replace the conventional separation method since its separation is based on molecular size, modest energy, and modular equipment requirement [2,3]. The capability of membrane separation used in the treatment of wastewater containing organic pollutant cannot be denied. Yet, enhancing the capability of the polymeric membrane in wastewater treatment for an organic pollutant is one of the latest technologies deeply discussed by researchers.

Phenolic compounds such as phenol are one of the most common organic water pollutants found in wastewater [4]. In the field of environmental research, many agree that phenol study is pertinent because the selection of phenol as a model pollutant has been widely explored and the availability of phenol removal and destruction data with respect to wastewater treatment is effortlessly obtained [5,6]. Among various advanced treatment techniques that involve phenol such as Fenton process, ozonation, and oxidation, photocatalytic has become one of the popular techniques being commercialized in recent years [7,8]. The photocatalytic process discovered more than 40 years ago has great potential as a low cost, environmentally friendly, and sustainable treatment technology to align with the zero-waste scheme in the wastewater treatment industry [9,10]. The ability of this advanced oxidation processes (AOP) has been demonstrated to remove organic compounds and microorganisms in the water by oxidation mechanism which depends on hydroxyl radical formation (·OH) [11].

In photocatalytic, selection of the right catalyst is an important part of ensuring the oxidation process takes place. Photocatalytic performance strongly depends on the semiconductor’s absorption capacity towards sunlight and the reaction efficiency of photo generated charge carriers [12]. Improving the use of visible light energy and preventing the recombination of electron–hole pairs are two current concerns in the selection of active photocatalysts [13]. Band gap engineering via extrinsic modifications enables larger visible light absorption relative to self-induced intrinsic modification in photocatalyst. Various semiconductors have been tested for their efficiencies towards phenol degradation [14]. Among those photocatalysts, graphitic carbon nitride (g-C_3_N_4_ or GCN) is regarded to be the most stable allotrope at ambient conditions [15,16]. The great advantage of this catalyst as a metal-free compound is that it inherently allows the presence of functional group along with the reactants (e.g., carboxylic acids or alcohols) or leaving groups (e.g., OH or NH_2_), which usually passivates the metal catalyst [17]. Other than that, GCN can be facilely synthesized using inexpensive precursors (e.g., melamine or dicyandiamide), is active under visible light irradiation, and is chemically stable [18]. In addition, GCN has attracted worldwide attention due to its various excellent merits in response to visible light, high photochemical stability, and easily modified electronic structure [19]. Photocatalytic efficiency of GCN is still limited due to the large optical band gap (2.6 eV) which corresponds to utilisation of solar energy (*λ* < 460 nm) and the fast recombination rate of photo generated electron–hole pairs [13].

An ideal semiconductor photocatalyst should feature an excellent light absorption ability, effective charge separation, and long-time stability [20]. Therefore, any alteration of GCN requires the enhancement of the three aforementioned properties to improve the photocatalytic performance [21]. Researchers have made numerous efforts to enhance the properties of GCN as photocatalyst by applying heterojunctions with other catalysts. The doping of foreign elements such as oxygen into the matrix structure can transform the textural structure, chemical composition, atomic arrangement, physical dimensions (carrier limit), electronic properties, and band position of the photocatalyst, resulting in different capabilities to capture photons and separate the electron–hole pairs. Besides that, this technique is able to change the electronic properties by manipulating the multiple defects and distortion into a semiconductor system [22].

Oxygen doping technology could be used to change the properties of GCN for higher efficiency and wide application [23]. This technology is also able to influence multiple defects and distortion into a semiconductor system leading to the changes of electronic properties [24]. Photoactivity oxygen doping for the graphitic carbon nitride is much higher than that for the pure GCN, and the boundary photoactivity OGCN in the visible spectrum ranges from 460 to 498 nm. Therefore, all modified GCN perform better degradation under visible light and sunlight radiation than pure GCN [25]. The purpose of oxygen doping is to increase the light radiation absorption and pollutant adsorption on GCN because the oxygen bearing groups suppress the recombination of electron–hole pairs. Oxygen modification can help to expand surface area, improving the physisorption and chemisorption, extending light absorption edge, and then increasing the photocatalytic activity [26]. The higher surface area of the catalyst, the more visible lights can be absorbed by the photocatalyst. Eventually, this will help to increase the photocatalytic process. High concentration of oxygen alerts the direct semiconductor into an indirect semiconductor, while the negative oxygen atom becomes the active centre for enhanced light harvesting [27]. The oxygen atomic radius is 48 ppm while the electronegativity of oxygen atom is 3.44 eV (in Pauling scale). High electronegativity of oxygen could limit electronic mobility and protect photo-generated holes without lattice distortions. Hence, oxygen is a good candidate for GCN modification semiconductor.

Regardless of the great potential offered, the photocatalytic technique that involves suspended photocatalysts into the wastewater requires posttreatment for catalysts purification before it can be reused [28]. These steps have become major drawbacks since they are not economically effective. The immobilisation of catalyst into the inert surface such as a polymeric membrane is thus foreseen as a promising solution. Even though catalyst embedded into the inert surface will limit the mass transfer of pollutants to the catalyst surface and reduce the photocatalytic activity, it will eliminate the posttreatment step and save costs. However, there are still no limitations to improve the performance of the catalyst immobilisation technique.

It is crucial to identify an effective membrane specially designed for the photocatalytic process to improve the photocatalytic immobilisation technique on the polymeric-based membrane. Besides the improvement of catalyst active surface area, the enhancement of phenol degradation is another main objective. Therefore, modification of membrane must be done to develop these two aspects. To overcome all mentioned issues, superlative hydrophilic surface modifying macromolecules (LSMM) was used in this study to increase the active surface area of the OGCN photocatalyst on the top surface of the PES membrane. The effect of OGCN photocatalyst loading on the hybrid photocatalytic membrane was further investigated in this study. The approach is supposed to increase the efficiency of the photocatalyst, hence improving the photocatalytic activity and overall water treatment processes.

## 2. Experimental Procedures

### 2.1. Materials

The commercial polyethersulfone (PES) powder was purchased from Solvay Veradel^®^, US. *N*-Methyl-2-pyrrolidone (NMP) (Analytical Grade, RCI Labscan Limited, Bangkok, Thailand) which was employed without further purification and was used as the main component for the membrane fabrication. In the preparation of oxygenated carbon nitride (OGCN), melamine (C_3_H_6_N_6_) was purchased from Sigma Aldrich (St. Louis, Missouri, USA) and hydrogen peroxide (H_2_O_2_, Analytical grade, 30%) purchased from Merck Millipore (Burlington, MA, USA). Meanwhile, hydrophilic surface modifying macromolecules (LSMM) were supplied by University of Jordan (molecular weight, MW, ~4050 g/mol) and phenol powder for the synthetic wastewater for photocatalytic testing was purchased from QRec (QRec, Chon Buri, Thailand).

### 2.2. Synthesis of Photocatalyst

**Figure 1:**
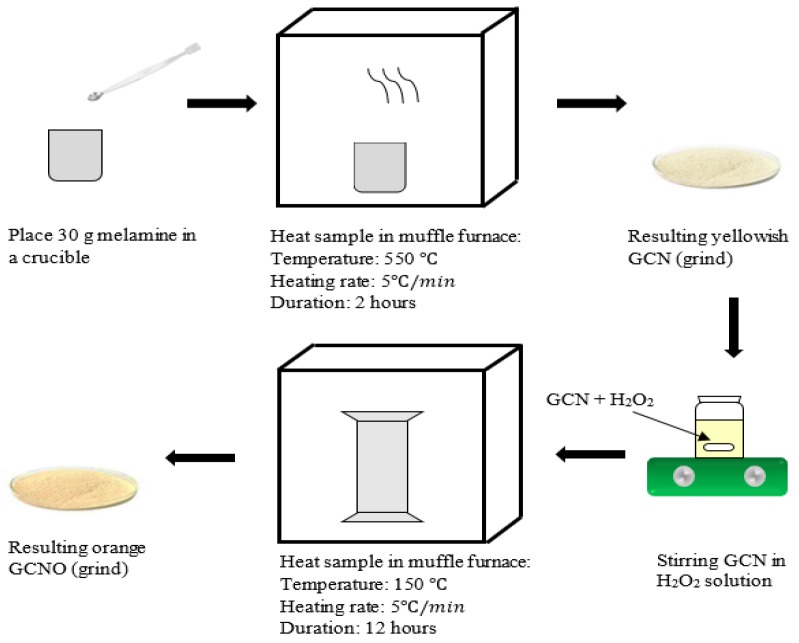
Synthesis procedure of the oxygen doped GCN (graphitic carbon nitride).

OGCN photocatalayst was synthesised according to our previous study [29]. The preparation of OGCN involved two stages which were the preparation of graphitic carbon nitride (GCN) by condensation method and hydrothermal treatment using H_2_O_2_ for OGCN production. First, 30 g of melamine in a half-opened crucible boat was heated in a muffle furnace at 550 °C for 2 h under 5 °C/min heating rate. A yellow powder of GCN was formed and was subsequently ground using an agate mortar to produce fine powders. Next, 1 g of GCN was mixed with 50 mL of H_2_O_2_ solution in 120 mL Teflon-lined stainless-steel autoclave and was vigorously stirred for 30 min. Subsequently, the autoclave was sealed tightly and placed in an oven at 150 °C for 12 h. The resulted orange powder was then washed using RO water and further dried in an oven at 60 °C to remove excess water. This product was named OGCN photocatalyst. The method to produce OGCN was illustrated as in Figure 1.

### 2.3. Preparation of PES/LSMM-OGCN Photocatalytic Membrane

The PES hybrid photocatalytic membrane was fabricated under single casting step by phase inversion technique. The effect of OGCN loading on the fabrication of PES/LSMM-OGCN membrane was studied. The constant parameter of LSMM loading at 4 wt% was selected as the optimum LSMM loading. First, 4 wt% of LSMM particles were dispersed in NMP and 18 wt% of PES polymers was added. The obtained polymer solution was used to prepare the reference PES membrane. The solution was then stirred overnight or until the polymer was fully dissolved. The dope solution was then ultrasonicated for 1 h to mix the material solutions well and labelled as PES/LSMM-OGCN0%. Then, the above steps were repeated with an addition of 0.5 to 2.5 wt% of OGCN with the increment of 0.5 wt%.

In the preparation of the photocatalytic membrane, 10 mL of dope solution was spread onto a glass plate to form a solution layer at a nominal thickness of 70–80 µm. A glass rod was used to spread the dope solution onto the glass plate to produce flat sheet membrane. After 5 min of solvent evaporation time, the plate was immediately immersed in RO water coagulation bath at room temperature and the membrane slowly formed a flat sheet membrane. After several minutes, the membrane self-detached from the glass plate. The immersion was left overnight to ensure complete solidification and removal of residual solvent from the membranes. After 24 h, the membrane was air dried at room temperature for 3 days and was ready to use.

### 2.4. Membrane Characterisation

The effect of OGCN loading on the fabrication of PES photocatalytic membrane was further analysed under various characterisation analyses. The morphology of the PES hybrid photocatalytic membrane was analysed using scanning electron microscope (SEM, JEOL IT 300LV, Tokyo, Japan) on the cross section of the membrane. The sample was prepared by immersing the membrane sample in methanol first and then quickly soaking it in liquid nitrogen for approximately 30 s; it was then fractured using a sharp knife to obtain a clean break. Meanwhile, the membrane surface morphology was analysed using a table top scanning electron microscope (SEM, TM3000 Hitachi High Technologies America, Tarrytown, NY, USA). Prior to the analysis, all membrane samples were coated with platinum under vacuum for about 5 min to obtain a clear image. Meanwhile, the surface topography and roughness of the top membrane were investigated using atomic force microscope (AFM) with tapping mode AIST-NT (SPA400 DFM, Seiko, Tokyo, Japan). Prior to AFM analysis, a small piece of each membrane was cut and glued on the top of a 1 cm^2^ round sample holder. The surface roughness of the membrane was measured using AIST-NT SPM Control software (SPA400 DFM, Seiko, Tokyo, Japan).

### 2.5. Physical Characteristics of the Hybrid Photocatalytic Membrane

The physical characteristics of the prepared membrane were further analysed on the basis of membrane hydrophilicity, pure water flux, and phenol rejection. The relative hydrophilicity of the prepared membrane was determined by measuring the contact angle on the top and bottom membrane surfaces using sessile drop method with goniometer (OCA15Pro, Data Physics, San Jose, CA, USA). The measurement was done by dropping a water droplet (0.3 µL) at a dosing rate of about 0.5 µL/s on the flat membrane using a microsyringe. The water droplet was dropped on 10 different spots of the membrane samples and the average contact angle was calculated. The contact angle measurement was performed on both top and bottom surfaces of all the membrane samples.

The pure water flux experiment was conducted using crossflow filtration system at 4 bar at room temperature. The prepared hybrid photocatalytic membranes were pre-pressured to minimise its compaction effect using deionised water for 30 min at a pressure of 2 bar before measurement and was subsequently measured at 1 bar. The pure water flux, *J_w_* (L/m^2^·h) of the membrane was calculated using Equation (1):

(1) $$J_{w}=\frac{V}{A\;\times \;\Delta t}$$

where *V* is the permeate liquid (L), *A* is the effective membrane area (m^2^), and Δ*t* is the time of permeate collection (h).

The phenol rejection analysis was conducted through a continuous crossflow water permeation for flat sheet module system. Two litres of water containing 10 ppm of phenol was used as a feed. The concentration of phenol in the feed and permeate were characterised using a UV–visible (UV–vis) spectrophotometer (DR5000, Hach, Loveland, CO, USA) at 294 nm, at which the maximum absorption occurs. The phenol rejection was expressed in the percentage of rejection according to Equation (2):

(2) $$\;R\left(\%\right)=\left[1-\left(\frac{C_{ph,p}}{C_{ph,f}}\right)\right]\;\times \;100$$

where *C_ph_*_,*p*_ and *C_ph_*_,*f*_ are the concentrations of phenol in the permeate (ppm) and feed (ppm), respectively.

### 2.6. Adsorption and Photocatalytic Activity Measurement

The fabricated membranes were tested for adsorption of phenol on the membrane surface to study their adsorption capacity. This experiment investigated the time required to achieve adsorption–desorption equilibrium. Furthermore, this experiment is essential to ensuring that further phenol degradation is contributed by photocatalysis, thus differentiating the exact role of adsorption and photocatalysis. Membrane samples were attached to a water chamber module and stirred for 90 min under dark condition. 1 L of 10 ppm phenol solution was used in this experiment. Solution samples were collected every 10 min to determine the time required for maximum adsorption by the membrane. The sample was then analysed using a HPLC with a UV detector (Model G4288C, Agilent Technology, Santa Clara, CA, USA). The time for full adsorption was determined when a stable reading was achieved.

The efficiency of hybrid photocatalytic membranes was evaluated on the basis of phenol degradation. In this study, the fabricated PES/LSMM-OGCN membranes were tested for phenol reduction in aqueous solution. An ultraviolet (UV) lamp (Vilber Laurmat, *λ* = 312 nm, 30 W, light intensity 3.0 mW/cm^2^) and a visible lamp (light-emitting diode (LED) lamp, *λ* = 420 nm, 30 W) were used as the light sources in the experiment. The chamber was placed under the light sources with a 15 cm gap from the light to the membrane. Continuation from the adsorption–desorption for 120 min, the membrane sample was exposed to the light sources for the photocatalytic degradation processes. Five millilitres of the phenol solution samples were collected using a syringe every 30 min. The samples were exposed to light (UV and visible) for 300 min. To determine the photocatalytic degradation efficiency, the phenol degradation (%) was calculated using Equation (3):

(3) $$\;\mathrm{Phenol}\;\mathrm{degradation}\;\left(\%\right)=\left[\frac{\left(C_{f,t}-C_{p,t}\right)}{C_{f,t}}\right]\;\times \;100\%$$

where *C_f_*_,*t*_ and *C_p_*_,*t*_ are the phenol concentrations in the initial feed (ppm) and permeate (ppm), respectively.

## 3. Results and Discussion

### 3.1. Oxygenated Graphitic Carbon Nitride (OGCN) Characterisations Study

**Figure 2:**
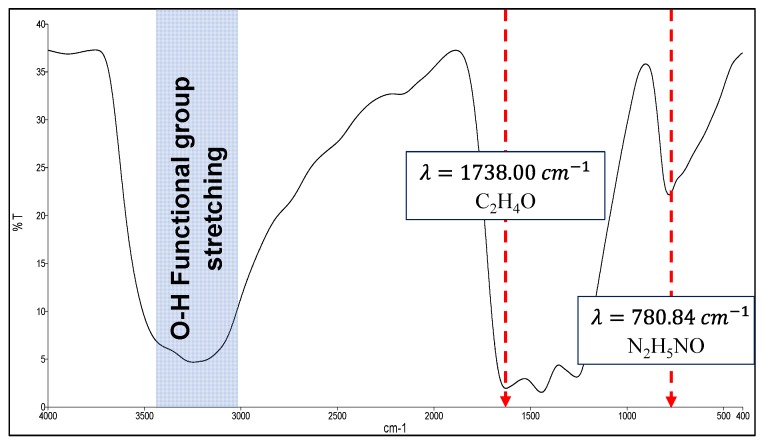
FTIR spectrum of OGCN (oxygenated graphitic carbon nitride) obtained in this study.

The synthesised OGCN was characterised via FTIR and OCHNS analyses to confirm that it was successfully oxygenated. The FTIR analysis was conducted to identify the formation of oxygen-containing functional groups in OGCN. Figure 2 shows the FTIR spectrum of OGCN. Two peaks appeared at 771 and 1728 cm^−1^, corresponding to the N_2_H_5_NO and C_2_H_4_O compounds. The attained spectrum was comparable with the OGCN spectrum reported by Liu et al. [22].

The synthesised OGCN was analysed by CHNOS analyser to quantitatively measure the percentage of C, H, N, O, and S elements in OGCN. The analysis successfully quantified the percentage of elements as tabulated in Table 1. The low percentage of S compound might come from the ashes resulted from the burning method of the CHNOS analyser. A comparable percentage of the elements in OGCN with Liu et al. [22] was obtained. Since the oxygenation process in this study followed the method provided by Liu et al. [22], it is worth comparing the obtained CHNOS values with their results [22].

### 3.2. Confirmation of the Successfulness of OGCN and LSMM Incorporation in PES Matrix

**Figure 3:**
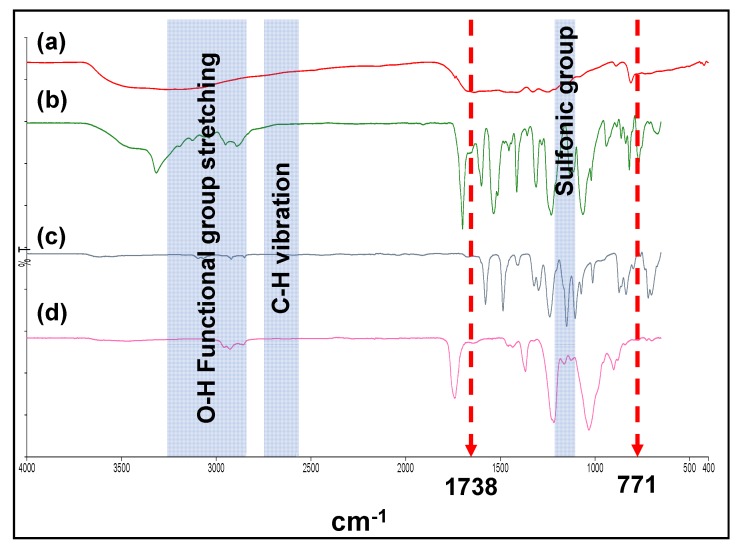
FTIR spectra for (a) OGCN catalyst particle; (b) LSMM (hydrophilic surface modifying macromolecules) additive; (c) PES18wt% membrane; and (d) PES/LSMM-OGCN membrane.

The FTIR analysis for the neat PES polymer-based membrane, synthesised OGCN, and LSMM additive was carried out to study the changes in functional group in the fabricated PES/LSMM-OGCN membranes and the spectra are shown in Figure 3.

Figure 3 shows that both OGCN and LSMM have the O–H stretching vibration which are assigned between 3500 and 3100 cm^−1^. The peaks that appeared between the ranges corresponded to the alcoholic and phenolic hydroxyl groups of the chemical compounds [30]. By investigating the OGCN spectra of oxygen functional group at 771 and 1738 cm^−1^, it was found that the peaks disappeared after the OGCN was incorporated into the membrane. There are some noticeable changes to the FTIR spectra of the resulted PES/LSMM-OGCN membrane. The peaks at 1350–1450 cm^−1^ corresponded to the sulfonic group at the end and capped off the LSMM molecular structure and were observed to be shifted to a longer wavelength for the ES/LSMM-OGCN membrane sample. Less OH functional group of LSMM was observed in the ES/LSMM-OGCN membrane. This proves that there was a hydrogen interaction between OGCN and LSMM. Benefiting from the migration properties of LSMM, the anchored LSMMOGCN could successfully move the OGCN to the membrane top surface.

### 3.3. Morphology Analysis of PES/LSMM/OGCN Membrane

**Figure 4:**
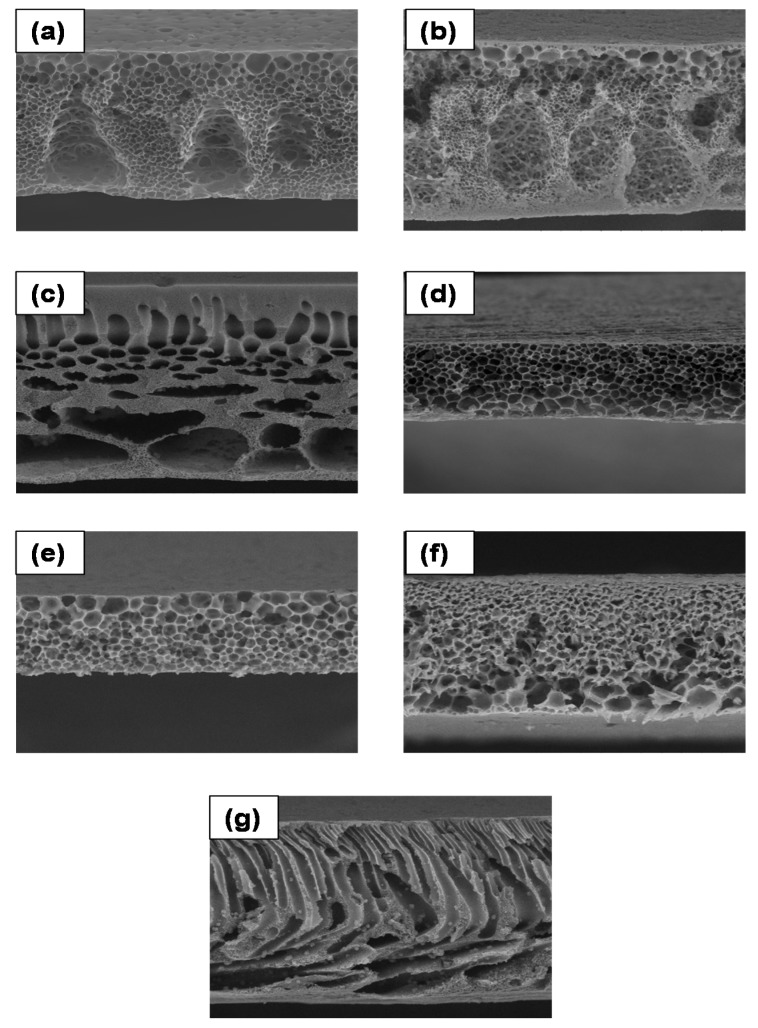
SEM images of cross section of PES/LSMM/OGCN membranes at 1000× magnification: (**a**) PES18wt%; (**b**) PES/LSMM-OGCN0wt%; (**c**) PES/LSMM-OGCN0.5wt%; (**d**) PES/LSMM-OGCN1.0wt%; (**e**) PES/LSMM-OGCN1.5wt%; (**f**) PES/LSMM-OGCN2.0wt%; and (**g**) PES/LSMM-OGCN2.5wt%.

Figure 4 shows the SEM images of the cross section of membranes at 1000× magnification. The SEM images in Figure 4a–c for PES18wt%, PES/LSMM-OGCN0wt%, and PES/LSMM-OGCN0.5wt% membranes, respectively, show that the membrane body consisted of a tear- and sponge-like structure. The structure was produced because of the instantaneous phase inversion that took place between solvent and nonsolvent in the coagulation bath. Besides, the strong affinity between NMP and water which was used as the solvent and coagulant also caused the formation of such a structure [31]. However, as shown in Figure 4c, the tear-like structure of PES/LSMM-OGCN0.5wt% started to diminish. As the OGCN loading was increased, the membrane structure formed a completely sponge-like structure. According to our previous study, the microvoid sponge-like structure formed due the formation of the structure type contributed by the solvent evaporation time of 5 min [29]. The findings conclude that the presence of OGCN in PES/LSMM-OGCN membrane assisted in vanishing the tear-like structure of the membrane to become a sponge-like structure. 

**Figure 5:**
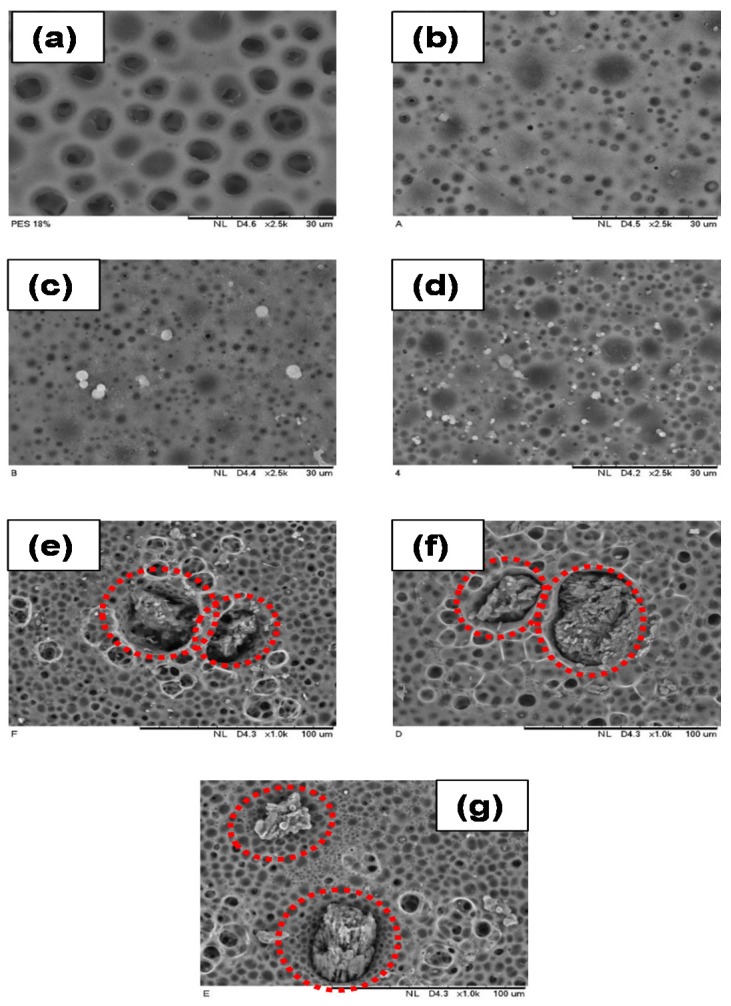
SEM images of top surface of PES/LSMM-OGCN membranes at 2500× magnification: (**a**) PES18wt%; (**b**) PES/LSMM-OGCN0wt%; (**c**) PES/LSMM-OGCN0.5wt%; (**d**) PES/LSMM-OGCN1.0wt%; (**e**) PES/LSMM-OGCN1.5wt%; (**f**) PES/LSMM-OGCN2.0wt%; and (**g**) PES/LSMM-OGCN2.5wt%.

Figure 5 shows the SEM images of the top surface morphology of all the PES/LSMM-OGCN membranes at 2500× magnification. Figure 5a,b shows no white particles as no OGCN was added in the membrane. In Figure 5c,d, OGCN particles were well distributed on the PES surface as the loading was increased up to 1.0 wt%. PES18wt% membrane in Figure 5a had quite a number of open pores on the top surface. Open pores indicate the porous structure of the membrane surface. The open pores were formed due to higher counter diffusion velocity of solvent and nonsolvent, and resulted in membranes with high porosity and pore size, especially at the membrane surface. Besides, interfacial pores were also formed as a consequence of shrinkage at the polymer phase during the de-mixing process due to interfacial stress between the polymer and catalyst particles [32]. A membrane with porous structure is good for wastewater application as it could increase the water flux performance. However, if the pore size of the membrane is larger than the size of phenol molecules, it will not benefit the separation process as the phenol molecules could escape to the permeate side. Eventually, the addition of LSMM in PES membrane as shown in Figure 5b helped to reduce the volume of open pores on the PES membrane top surface.

The existence of OGCN particles was detected on the membrane top surface for the catalyst added membrane. As the OGCN loading was increased from 0.5 to 1.0 wt%, more white particles could be seen on the membrane top surface. It can be concluded that the distribution of the particles improved as the OGCN loading was increased. However, the agglomeration of the catalyst started to occur at 1.5 wt% of OGCN loading (Figure 5e). The agglomeration was caused by the effect of van der Waals force. As the amount of OGCN was increased, the attraction of intermolecular forces between molecules became stronger and led to agglomeration in the composite membrane. Besides, the fine size of OGCN particles together with their large surface area to volume ratio and surface energy were the other reasons for agglomeration to occur during the process [22]. Agglomeration is not good for wastewater application since it reduces the lifespan of the membrane and possesses a low active surface area to degrade the pollutant [33]. Other than that, as shown in Figure 5e–g, the open pores occurred on the membrane where agglomeration occurred. The presence of small aggregated OGCN particles at 1.5, 2.0, and 2.5 wt% of OGCN loadings at the membrane top surface may interrupt the polymer chain packing and resulted in the open pores. Open pores could be advantageous to increase the water flux amount. However, it might not be beneficial to the rejection result. Larger open pore size on membrane top surface increases the tendency of smaller sized organic pollutant particulates to escape to the permeate side.

### 3.4. Surface Roughness Analysis of PES/LSMM/OGCN Membrane

**Figure 6:**
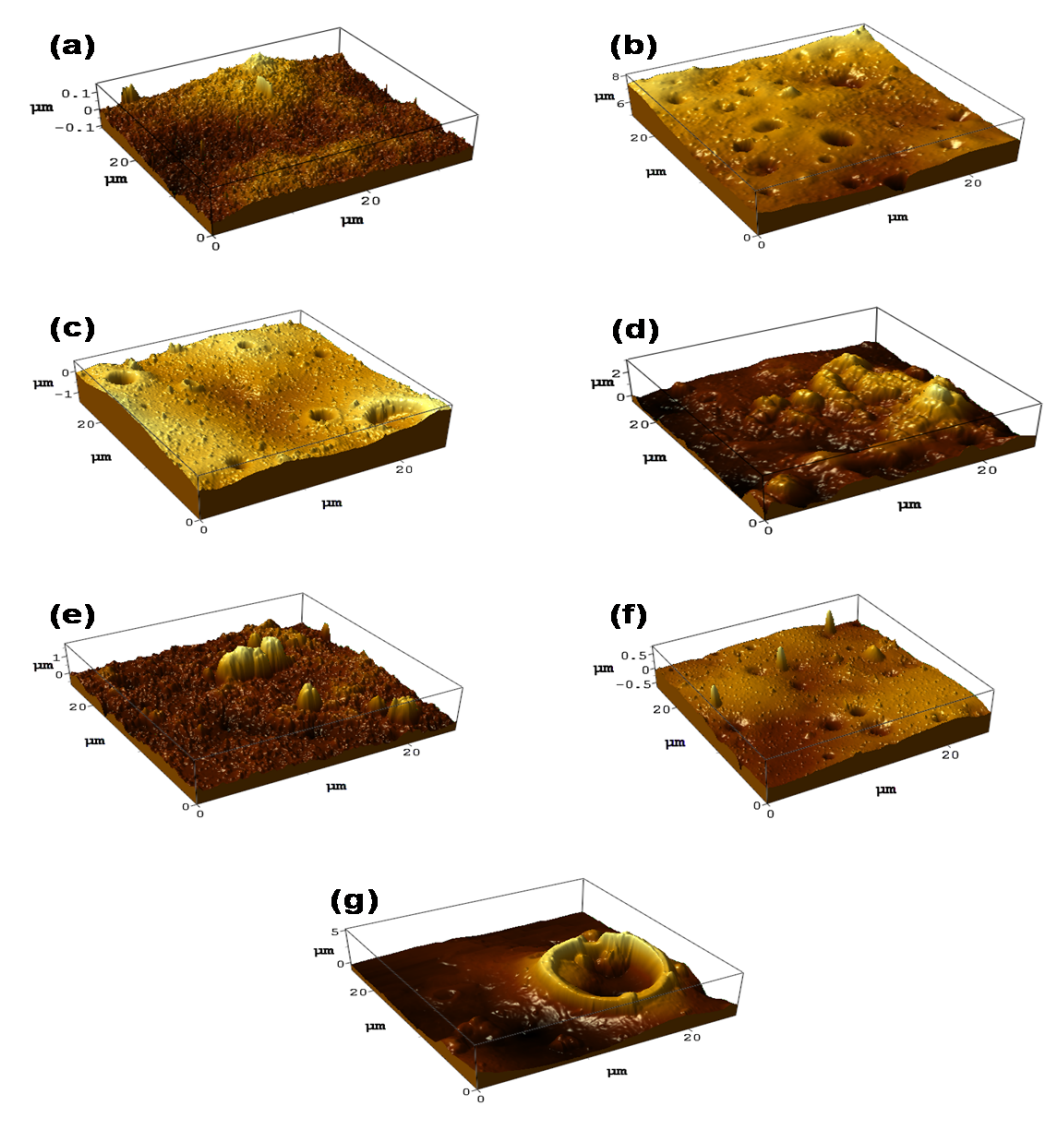
AFM (atomic force microscope) images of PES/LSMM-OGCN membranes: (**a**) PES18wt%; (**b**) PES/LSMM-OGCN0wt%; (**c**) PES/LSMM-OGCN0.5wt%; (**d**) PES/LSMM-OGCN1.0wt%; (**e**) PES/LSMM-OGCN1.5wt%; (**f**) PES/LSMM-OGCN2.0wt%; and (**g**) PES/LSMM-OGCN2.5wt%.

Figure 6 displays the three-dimensional (3D) AFM images of the PES/LSMM-OGCN membrane surfaces, while Table 2 tabulates the surface roughness value based on the AFM analysis program. Roughness is important to determine the membrane interaction with pollutant in the treatment process. An addition of filler into PES membrane enhances the membrane surface roughness [34,35]. However, the roughness of PES/LSMMOGCN0wt% membrane without OGCN loading was lower than the roughness of the unmodified membrane. Hence, increasing the OGCN to 0.5 wt% abruptly increased the membrane roughness. According to the R_a_ values for the 0.5–2.0 wt% of OGCN loadings in Table 2, the highest roughness value was attained by the PES/LSMM-OGCN1.0wt% membrane. Nevertheless, further increase of the filler loading gave fluctuate reading of surface roughness. There are two possibilities for the trend of membrane surface roughness in this study. First, the roughness parameters depended on the Z values. Two aspects in expecting the roughness on membrane surface are the deep depressions which correspond to the pores and high peaks that describe the nodule. When the 3D images of AFM analysis were compared to the SEM top surface images, the PES/OGCN-LSMM1.0wt% membrane seemed to have the highest visibility of nodule representing OGCN. A good distribution of catalyst on the membrane surface aided by the LSMM increased the membrane surface roughness and promoted the photocatalyst surface area to be exposed to lights. Higher catalyst surface area is believed to have higher photocatalytic efficiency [36]. However, further increase of the catalyst loading caused a reduction of surface roughness and particle agglomeration, which gave a fluctuate value between PES/LSMM-OGCN1.5wt%, PES/LSMM-OGCN2.0wt%, and PES/LSMM-OGCN2.5wt% membranes. Roughness value also depends on the pore size where the value is proportional to the surface pores [37]. The agglomeration reduced the roughness due to the uneven distribution of catalyst within the scanned area. However, the PES/LSMM-OGCN2.5wt% recorded the highest surface roughness value with 311.0 nm. The formation of open pores on the membrane surface promoted by this agglomeration contributed to the high roughness value. This can be seen in Figure 6g where a large open pore crater- like image clearly appeared.

### 3.5. Hydrophilicity Analysis of PES/LSMM-OGCN Membrane

**Figure 7:**
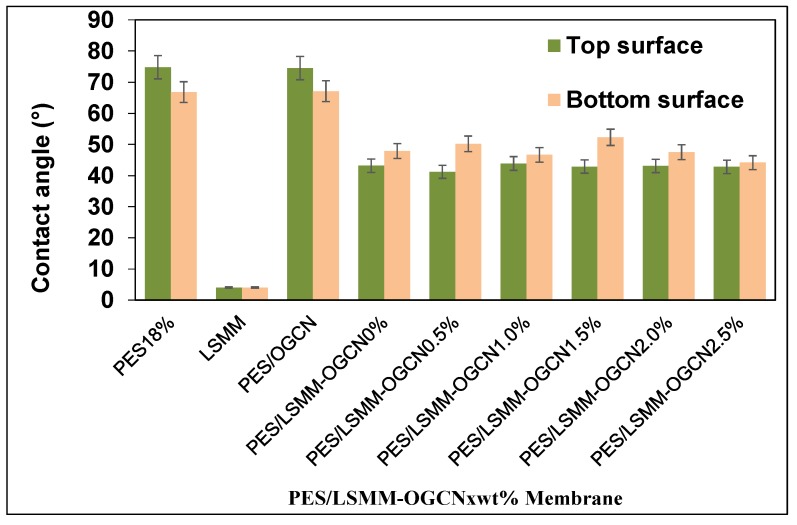
PES/LSMM-OGCN membrane contact angle.

In wastewater treatment, the application of polymeric membrane is to separate and filter the contaminant in the water. Therefore, the polymeric membrane should have a good hydrophilic characteristic to function well in wastewater treatment. However, PES itself has low hydrophilic properties. The disadvantage of hydrophobic or low hydrophilic polymer membrane is that they tend to have a fouling problem, especially in the water filter application. As can be seen in Figure 7, the presence of LSMM had a significant impact on the contact angle results. The contact angle value for the top and bottom surfaces of the PES membrane was 74.8° and 66.8°, respectively, which show that LSMM itself is super-hydrophilic. The addition of LSMM in PES/OGCN membrane reduced the contact angle values for the top and bottom surfaces of the membrane. On the basis of the measured contact angle, these different types of membrane show their hydrophilic characteristic since the contact angle was always below 90°. The contact angle for the top LSMM membrane surface was in the range of 41° to 44° as the content of OGCN was increased in the membrane composition, while the contact angle for the bottom LSMM membrane surface increased in the range of 44° to 52°. The addition of LSMM tended to minimise the interfacial energy of the mixed matrix. Therefore, LSMM with the lowest hydrophilic surface energy migrated and focused at the air interface. Hence, it helped to reduce the values of contact angle for the membranes. However, the hydrophilicity was not affected by the addition of OGCN particle immobilised in the PES/LSMM membrane as the result was within ±5% acceptable error. Commonly, when the hydrophilic substance is added into the dope solution, it will lead to an accelerated solvent and nonsolvent exchanges caused by thermodynamic immiscibility, which therefore encourages the formation of a more porous structure [38].

### 3.6. Pure Water Flux and Phenol Rejection Analysis of PES/LSMM-OGCN Membranes

Table 3 shows the relationship of water flux against the OGCN amount immobilised in the membrane. Generally, the permeation water flux increased as the amount of OGCN was increased in the dope solution and reached the maximum value of 300 L/m^2^·h when the OGCN amount was 1.5 wt%. Based on the result, the aggradation of OGCN particles formed a loose membrane layer and caused the low resistance of water permeation. A high porosity formed on the top surface of the membrane affected the flux through the membrane layer which became a little dense since the loading of OGCN was very small. This hindered water to permeate through the membrane. However, at the high loading amount of OGCN (2.0 wt%), the permeation water flux eventually decreased after it reached the maximum value at 1.5 wt% of OGCN loading. This is due to the precipitation of the catalyst through casting and phase inversion process that clogged the membrane pore and increased the resistance [39]. Normally, a sponge-like structure will offer a small resistance to water and results in low water resistance [40]. At the 1.5–2.0 wt% loading of OGCN, it will cause an increase in viscosity that leads to delayed de-mixing with a sponge-like structure [41]. However, large aggregated OGCN particles were present at the top and bottom surfaces of the membrane for 2.0–2.5 wt% loadings (Figure 5). The reduction of membrane porosity may be contributed by the clogged OGCN particles in the pores.

Table 3 also shows the result of phenol rejection. The PES/LSMM-OGCN1.0wt% membrane indicated the highest phenol rejection compared to other membranes. This is due to the higher roughness of membrane surface that could help to increase the efficiency of the filtration process [42]. Besides, the membrane with tear- and finger-like structures for 2.5 wt% of OGCN loading also had low water resistance resulting in high permeability compared to the membrane with sponge-like structure. However, 0.5 wt% of OGCN loading yielded a membrane with lower phenol rejection, even though it has the tear- and finger-like structures that could enhance the membrane filtration. This is due to the dense layer of the membrane, which influenced phenol rejection. Referring to the membrane with sponge-like structure for 0, 1.0, 1.5, and 2.0 wt% of OGCN loadings, PES/LSMM-OGCN1.0wt% had the highest phenol rejection compared to the other membranes with sponge-like structure. Interfacial pores on the PES/LSMM-OGCN1.0wt% membrane led to high phenol filtration. Additionally, the agglomeration existed on PES/LSMM-OGCN membranes with 1.5, 2.0, and 2.5 wt% of OGCN caused open pores to occur on the membrane skin layer. The open pores could increase the membrane flux but cause lower phenol rejection.

### 3.7. Photocatalytic Activity of PES/LSMM-OGCN Membrane

#### 3.7.1. Adsorption Analysis of PES/LSMM-OGCN Membrane

**Figure 8:**
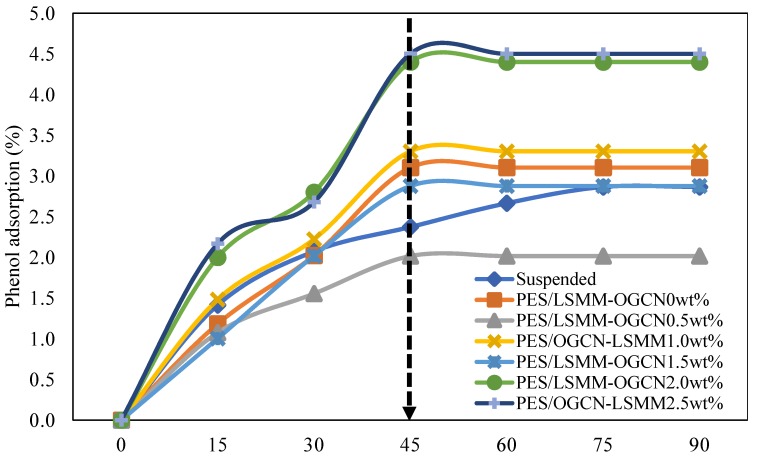
Phenol adsorption test using PES/LSMM-OGCN membranes.

The membrane adsorption capacity of phenol was determined on the basis of phenol adsorption using different loadings of OGCN in the PES/LSMM membranes (Figure 8). The PES/LSMM-OGCN0.5wt% membrane had the lowest phenol adsorption compared to the other membranes. The low adsorption is due to the dense surface layer of the tear-e and finger-like structures. The small amount of OGCN slowed down the precipitation rate and led to denser skin layer, thus reducing the filtration efficiency. However, the PES/LSMM-OGCN2.5wt% membrane had a good phenol adsorption due to the tear- and finger-like characteristics that led to low water resistance, thus helping to increase the filtration efficiency. The maximum value of phenol adsorption was 4.5% when the OGCN loading was 2.5 wt%. The sponge-like membrane tended to have small water resistance. However, it helped to improve the adsorption process.

#### 3.7.2. Photocatalytic Degradation of Phenol by PES/LSMM-OGCN Membrane

**Figure 9:**
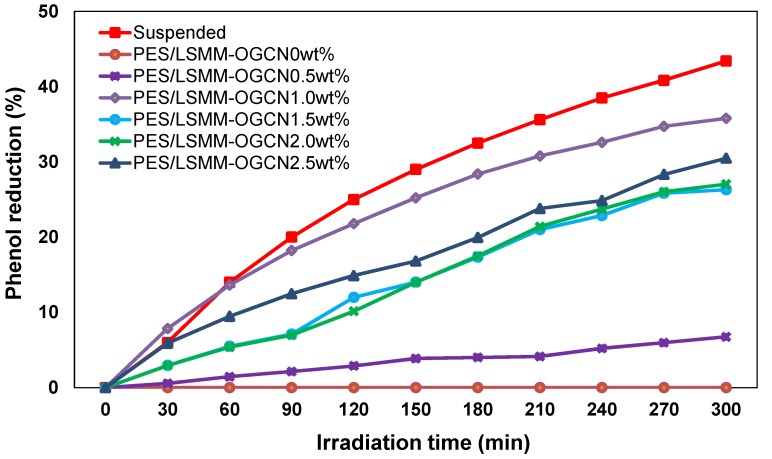
Phenol reduction via photocatalytic oxidation versus irradiation time for PES/LSMM-OGCN membrane.

The photocatalytic activity of OGCN loading on PES/LSMM membrane for phenol degradation is presented in Figure 9. There was no photocatalytic activity occurred for the PES/LSMM-OGCN0wt% membrane. Photocatalytic activity was signified by the phenol concentration reduction that was achieved after the OGCN loading at 0.5 wt%. These results confirm that the photocatalysis was initiated by the OGCN photocatalyst. For 0.5 wt% of OGCN loading on PES/LSMM-OGCN membrane, the low phenol degradation was due to the small loading of OGCN which provided low catalytic active surface area. The low loading of OGCN reduced the chances of UV light absorption capacity on the catalyst surface. Suspended catalyst method gives the highest phenol reduction via photocatalytic oxidation with percentage of phenol being reduced 43.4% within 5 h. This result is comparable to the data by Liu, 2016 [22]. The PES/OGCN-LSMM1wt% membrane gave the highest amount of phenol reduction among the fabricated membrane with 35.784 ± 0.5% phenol degradation. The photocatalytic phenol degradation was observed to be lower when the OGCN loading was increased to 1.5, 2.0, and 2.5 wt%. This was attributed to the low active surface area of the photocatalyst on the membrane since the catalyst tended to be agglomerated in the polymer matrix (Figure 9). The higher appearance of OGCN on the membrane top surface defined higher active surface area as explained in Figure 5. The agglomeration led to the low functional surface area and hence blocked the exposure of the photocatalyst to the UV light [43]. The performance of the photocatalytic activity for the membrane with 1.0 wt% of OGCN was good since it had the best particle distribution and higher volume of the active surface area on the membrane interface. Thus, the light absorption capacity also increased and helped to increase the photocatalytic performance.

## 4. Conclusions

PES/LSMM-OGCN hybrid photocatalytic membranes were successfully fabricated via the phase inversion technique in flat sheet form at ambient temperature. The OGCN loading was studied and no effect was observed on the membrane hydrophilicity on the basis of contact angle analysis. The SEM analysis revealed that OGCN nanoparticles agglomeration was formed at the OGCN loading higher than 1.0 wt%. The distribution of OGCN on the membrane surface was well distributed at low loading, while it would start to agglomerate at 1.5 wt% of OGCN. The best amount of OGCN in the casting solution was 1.0 wt% as the agglomeration did not occur, considering the stability of membrane performance and morphology. Agglomeration may reduce the catalyst surface area provided for photocatalytic activity. The highest flux was achieved by the PES/LSMM-OGCN1.5wt% membrane with 264 L/m^2^·h. However, the highest flux performance was not an advantage in this situation as the flux reduced the rejection value due to open pores. The highest rejection was achieved by the PES/LSMM-OGCN0wt% membrane with 15.41% of phenol rejected. The membrane with the highest photocatalytic performance was obtained at 1.0 wt% of OGCN loading with 35.78% phenol degraded after 6 h. Further modification such as surface modification by plasma etching, thin film coating, chemical grafting, and polymer blending with nanomaterials can be carried out to fine-tune the membrane pore size according to the phenol size (0.57 × 0.43) nm for better rejection performance. Regardless of the lower rejection value, the performance shown by the PES/LSMM-OGCN1.0wt% membrane was still competent because of the small difference of less than 1% to that of the PES/LSMM-OGCN0wt% membrane. On the basis of membrane performance evaluation, 1.0 wt% of OGCN loading was the most ideal amount that provided superior OGCN distribution and the highest phenol photocatalytic activity and selectivity without disrupting the membrane and catalyst state.

## Figures and Tables

**Table 1:** CHNOS elemental analysis of OGCN.

Element	C	H	N	O	S
Percentage (%)	57.5938	2.1968	31.6516	8.0082	<1

**Table 2:** PES/LSMM/OGCN membrane top surface roughness.

Membrane	Total Surface Roughness, R_a_ (nm)
PES18wt%	48.8
PES/LSMM-OGCN0wt%	30.8
PES/LSMM-OGCN0.5wt%	169.0
PES/LSMM-OGCN1.0wt%	463.1
PES/LSMM-OGCN1.5wt%	199.6
PES/LSMM-OGCN2.0wt%	101.0
PES/LSMM-OGCN2.5wt%	311.0

**Table 3:** Separation performances of PES/LSMM-OGCN membranes prepared at different OGCN loadings.

Membranes	Pure Water Flux, *J_w_* (L/m^2^·h)	Phenol Rejection (%)
PES18wt%	55.50	0.52
PES/LSMM-OGCN0wt%	17.6	15.41
PES/LSMM-OGCN0.5wt%	3.5	2.65
PES/LSMM-OGCN1.0wt%	32.2	14.73
PES/LSMM-OGCN1.5wt%	264.0	2.65
PES/LSMM-OGCN2.0wt%	106.5	3.53
PES/LSMM-OGCN2.5wt%	176.5	3.26
